# Quantitative assessment of fetal lung development using MRI-based radiomics: a comparative analysis of 3D and 2D approaches with external validation

**DOI:** 10.3389/fphys.2026.1920572

**Published:** 2026-07-29

**Authors:** Yaoxi Jiang, Pan Xiao, Song Peng, Weidong Fang

**Affiliations:** 1 Department of Radiology, The First Affiliated Hospital of Chongqing Medical University, Chongqing, China; 2 Department of Radiology, Chongqing Health Center for Women and Children, Women and Children’s Hospital of Chongqing Medical University, Chongqing, China; 3 Department of Oncology, The First Affiliated Hospital of Chongqing Medical University, Chongqing, China

**Keywords:** external validation, fetal lung development, magnetic resonance imaging, prenatal diagnosis, radiomics

## Abstract

**Objective:**

Fetal lung development is a critical determinant of neonatal health. This study aimed to externally validate and compare the performance of 3D versus 2D MRI radiomics models in quantifying fetal lung development. Gestational age estimation was utilized as a quantitative surrogate marker for this assessment in a large multi-institutional cohort with an independent test set.

**Methods:**

To compare 2D versus 3D radiomics, we analyzed 292 fetal MRI scans using T2-weighted coronal images. The cohort comprised an original dataset (n=252) and an independent test set (n=40) from two institutions, with gestational ages ranging from 28 to 38^+6^ weeks. For each case, 102 radiomics features were extracted from manually segmented 2D (tracheal bifurcation plane) and 3D (entire lung) regions of interest (ROIs). Gestational age estimation models were developed using support vector regression (SVR) and partial least squares regression (PLSR) and validated via regression metrics. Furthermore, intraclass correlation coefficients (ICCs) were calculated to evaluate the correspondence between 2D and 3D radiomic measurements.

**Results:**

Features extracted from 3D ROIs exhibited superior performance for gestational age estimation, achieving Pearson correlation coefficients (r) of 0.904 (SVR) and 0.902 (PLSR) in the original dataset, and 0.846 (SVR) and 0.861 (PLSR) in the independent test set. In contrast, 2D ROI-based models showed significantly lower accuracy. The median ICCs for the correspondence between 2D and 3D radiomic measurements were 0.379 (IQR: 0.179–0.554; range: 0.010–0.880) in the original dataset and 0.247 (IQR: 0.060–0.384; range: −0.024–0.721) in the test set.

**Conclusion:**

This study demonstrates that 3D MRI radiomics features outperform 2D features for gestational age estimation, serving as a quantitative surrogate for fetal lung development. These findings underscore the clinical potential of 3D radiomics in non-invasive prenatal assessment. Future studies incorporating larger cohorts and standardized imaging protocols are encouraged to further enhance the model’s robustness and clinical utility.

## Introduction

Fetal lung underdevelopment is a leading cause of neonatal respiratory disorders, such as respiratory distress syndrome (RDS), which significantly impacts neonatal morbidity and mortality (Mally et al., 2013; Kim et al., 2017). Consequently, the objective assessment of fetal lung development is critical for clinical management, particularly in determining delivery timing for high-risk pregnancies, to optimize neonatal outcomes.

Current methods for evaluating lung status—including ultrasound-based fetal growth parameters (Hadlock et al., 1985; Goldstein et al., 1988), gray-scale histogram width analysis (Serizawa and Maeda, 2010), and pulmonary circulation assessment by Doppler ultrasound (Moety et al., 2015)—offer value but possess inherent limitations in diagnostic accuracy and clinical applicability. Crucially, the established gold standard, amniocentesis with biochemical analysis, is invasive and carries a risk of complications. Recently, radiomics analysis of two-dimensional (2D) fetal lung ultrasound images has emerged as a promising non-invasive approach for evaluating lung developmental status (Cobo et al., 2012; Palacio et al., 2012; Bonet-Carne et al., 2015; Palacio et al., 2017; López Sánchez et al., 2018; Burgos-Artizzu et al., 2019; Flosi et al., 2020; Du et al., 2022; Zamarian et al., 2022). However, a fundamental limitation of these studies is the reliance on 2D imaging, which fails to capture the complex, heterogeneous three-dimensional (3D) microstructure of the entire fetal lung, potentially leading to incomplete or biased feature extraction.

Given the need for a more comprehensive assessment, fetal magnetic resonance imaging (MRI) presents an ideal solution. MRI techniques enable the acquisition of 3D images of the entire fetal lung, facilitating the use of 3D regions of interest (ROIs) for radiomic analysis. MRI also overcomes the limitations of ultrasound, such as signal interference from gas, bone, maternal fat, amniotic fluid, or unfavorable fetal positioning. Thus, the application of radiomics to 3D MRI data presents a promising avenue for the quantitative assessment of fetal lung development.

To this end, and recognizing that gestational age (GA) serves as a robust quantitative correlate for lung development, we aimed to compare the performance of 2D versus 3D radiomic models in estimating GA from fetal lung MRI scans. We hypothesized that 3D features, capturing the entire organ volume, would provide a more accurate and robust assessment than single-plane 2D features. To test this, we developed and validated models using both approaches, with a particular focus on external validation using an independent test set to verify generalizability.

Ultimately, this work seeks to establish a novel, non-invasive method for quantifying fetal lung development and to lay the foundation for assessing this developmental status in pathological cases where GA alone is unreliable.

## Materials and methods

### Participants inclusion

Fetal MRI data were retrospectively retrieved from the databases of two institutions between January 2020 and April 2023. Data from the first institution were utilized as the original dataset, whereas data from the second institution were designated as the independent test set to enable external validation. To ensure the study cohort represented normal fetal lung development, inclusion criteria were limited to cases indicated for non-pulmonary ultrasound-suspected abnormalities, such as ventriculomegaly, posterior fossa cistern enlargement, low conus medullaris position, or placenta previa. Gestational age (GA) was determined based on the last menstrual period and verified by first-trimester dating ultrasound (crown-rump length).

To ensure the cohort was representative of normal physiological lung development and to minimize potential confounding factors, the following exclusion criteria were applied: multiple pregnancies; incomplete fetal MRI datasets or insufficient clinical information; fetal organic lung lesions; chromosomal abnormalities; intrauterine growth restriction (IUGR); intrauterine infections; oligohydramnios; prenatal corticosteroid therapy; and maternal systemic conditions known to affect fetal growth, such as gestational diabetes, pre-eclampsia, thyroid disease, heart disease, liver or kidney disease, severe anemia, or intrahepatic cholestasis.

### Fetal MRI protocol

Scans were performed using 1.5T Philips Ingenia and 1.5T GE Signa Hispeed scanners with body matrix coils. Coronal single-shot fast spin-echo T2-weighted images were used with the following parameters:

Philips Ingenia:TR/TE= 8,254/120 ms; field of view= 303 × 303 mm; slice thickness= 4 mm; matrix= 256 × 256; flip angle= 90°.

GE Signa Hispeed:TR/TE= 1,800/90 ms; field of view= 400 × 400 mm; slice thickness= 6 mm; matrix= 256 × 256; flip angle= 90°.

### Data processing

**Figure 1:**
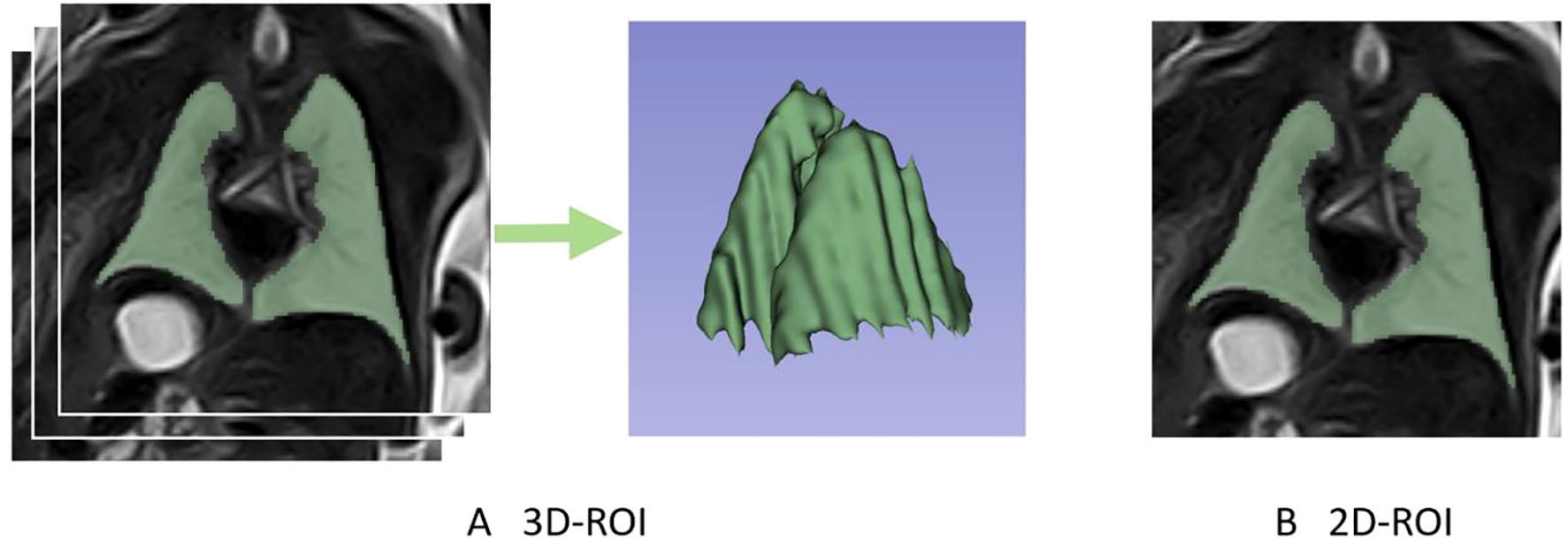
Representative coronal T2-weighted single fast spin-echo fetal lung MRI at 36 ^+ 2^ weeks of gestation. **(A)** 3D whole-lung ROIs generated using a semi-automated approach with manual refinement. **(B)** The corresponding 2D ROI, delineated in the plane of the tracheal bifurcation.

ROI delineation was performed collaboratively by four radiologists. Prior to delineation, all participating radiologists underwent standardized consensus training to collectively discuss and establish ROI boundary criteria (e.g., avoiding vessels, bronchi, and thymus). Subsequently, three radiologists with five years of fetal MRI diagnostic experience segmented the fetal lungs on coronal images using 3D Slicer (Fedorov et al., 2012), employing a semi-automated method (Level Tracing) with comprehensive manual correction. A subset of these initial, independent segmentations (n=25)was subsequently used to calculate the intraclass correlation coefficient (ICC) for assessing inter-rater reliability. A fourth radiologist with ten years of fetal MRI diagnostic experience reviewed and refined the segmentation results to generate 3D ROIs. The lung segmentation masks and MRI images were exported as NIFTI files. The slice index at the level of the tracheal bifurcation was recorded. Subsequently, the images of the tracheal bifurcation plane and the corresponding lung segmentation mask slices were converted to 2D NIfTI files using the Python package NiBabel (MIT). **Figure 1** illustrates the workflow for acquiring 2D and 3D ROIs.

Radiomics features were extracted using the Python package PyRadiomics (v3.0.1) (van Griethuysen et al., 2017) running on Python 3.7.6.Parameters included normalization (set to true), a voxel array shift of 300 (3 *SD*s × 100), and a bin width of 5. Interpolation was set to “sitkBSpline,” and resampled pixel spacing was specified as “[2,2,2]” for 3D data and “[2,2]” for 2D data. A total of 102 radiomics features were extracted, including 3D/2D shape (*n* = 9), first-order (*n* = 18), gray-level co-occurrence matrix (GLCM, *n* = 24), gray-level dependence matrix (GLDM, *n* = 14), gray-level run length matrix (GLRLM, *n* = 16), gray-level size zone matrix (*n* = 16), and neighborhood gray-tone difference matrix (NGTDM, *n* = 5).

### Model development and statistical evaluation

**Figure 2:**
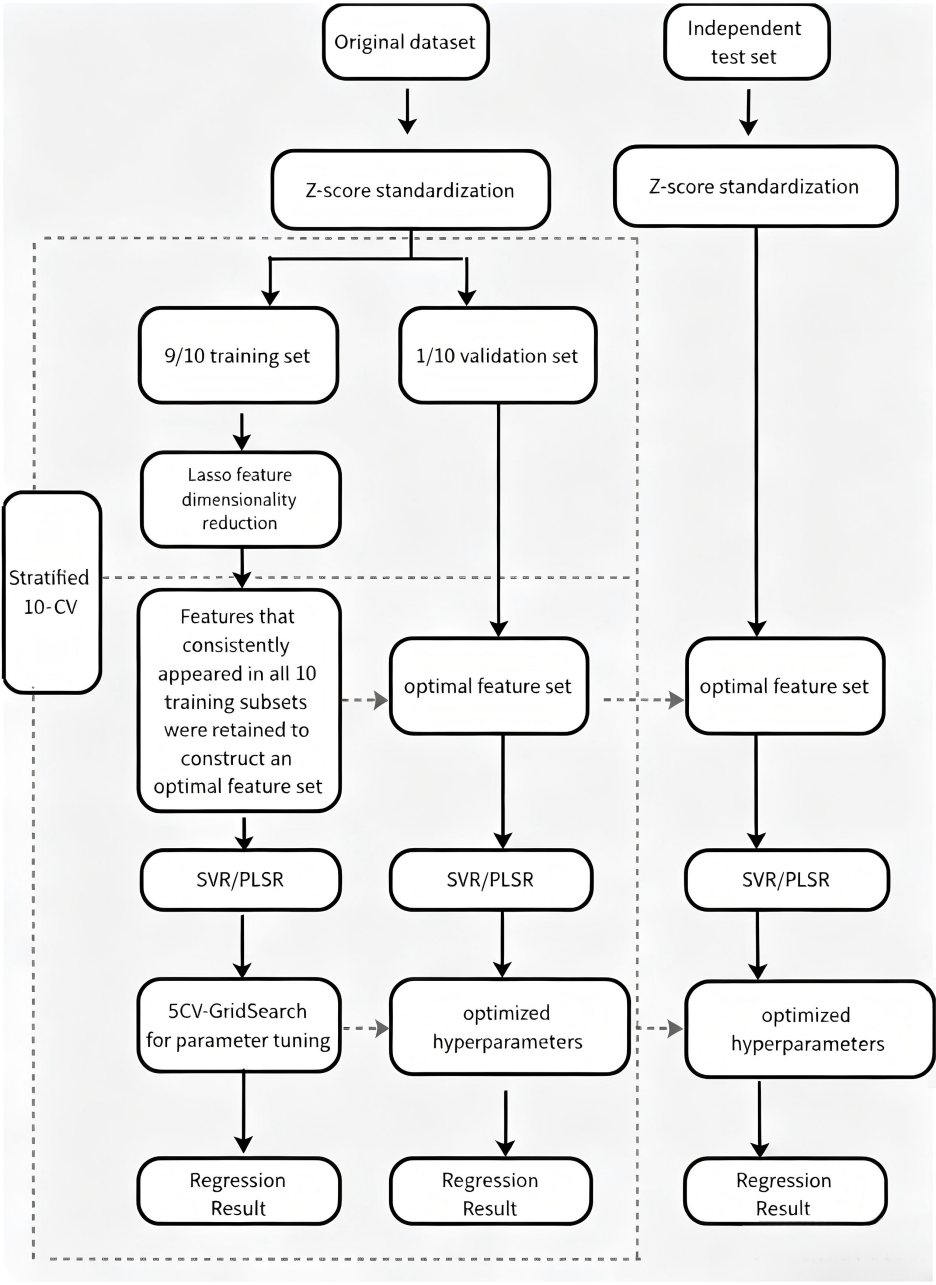
Flowchart of the model development and validation pipeline. The development process utilized a nested 10-fold cross-validation (CV) strategy. As illustrated, feature selection was performed using a stability selection approach, retaining only those features consistently selected across all 10 training folds to ensure robustness. Hyperparameter optimization was conducted via a nested 5-fold CV grid search. All feature selection and parameter tuning were performed exclusively within the training data of each CV iteration, strictly preventing data leakage.

The model development pipeline and validation strategy are illustrated in **Figure 2**. To ensure strict independence and prevent data leakage, all preprocessing and model development steps were performed using a nested procedure. First, data were processed using Z-score normalization; to prevent information leakage, the normalization parameters (mean and standard deviation) were calculated exclusively from the training subsets of each cross-validation iteration and then applied to the corresponding validation and independent test sets.

A stratified 10-fold cross-validation (CV) strategy was applied. The original dataset was randomly partitioned into 10 subsets, with nine forming the training fold and the remaining one serving as the validation fold in each iteration. To strictly avoid data leakage, all feature selection and hyperparameter tuning were nested within these training folds. Dimensionality reduction was conducted using the LASSO algorithm with the minimum *λ* criterion within each training fold. We employed a stability selection strategy: features that consistently appeared in all 10 training subsets were retained to construct an optimal feature set. For each feature in this optimal set, a stable importance weight was calculated as the mean of its regression coefficients obtained from the LASSO algorithm across all 10 folds.

Subsequently, support vector regression (SVR) and partial least squares regression (PLSR) models were utilized to estimate fetal gestational age. Statistical analyses were conducted using SPSS (v24.0). Hyperparameters for these regression algorithms were fine-tuned within the training subsets using a grid search strategy and 5-fold CV, which was strictly nested within the training loop (detailed search ranges are provided in **Supplementary Table 2**).In line with the cross-validation strategy, we implemented a stratified 10-fold CV model using a deterministic splitting approach (shuffle=False, random_state=None). This ensures that the inherent order of the fetal gestational age data is preserved across folds, providing a stable, reproducible, and clinically consistent evaluation framework. The optimized hyperparameters were then applied to evaluate the model on the held-out validation fold and, ultimately, on the independent test set.

Regression performance was evaluated using metrics such as the coefficient of determination (*R*^2^), adjusted-*R*^2^, mean absolute error (MAE), mean squared error (MSE), root mean squared error (RMSE), and Pearson correlation coefficients.

Descriptive statistics for the study population were reported as means with standard deviations or frequencies with percentages, as appropriate. Intraclass correlation coefficients (ICCs) were computed using Python to assess the agreement between 2D and 3D radiomic measurements in the original dataset and independent test set. A two-way mixed-effects model (ICC3) with a single rater unit was employed. The level of agreement was classified as excellent (ICCs >0.9), good (0.75 < ICC ≤ 0.9), moderate (0.5 < ICC ≤ 0.75), or poor (ICC ≤ 0.5) (Koo and Li, 2016).

## Results

The final study cohort comprised 292 subjects, with gestational ages ranging from 28 to 38^+6^ weeks. The original dataset included 252 cases from the first institution, while the independent test set comprised 40 cases from the second institution for external validation. Demographic characteristics and the distribution of cases across gestational weeks are detailed in **Tables 1**, **2**.

**Table 1:** Number of cases per gestational week.

Gestational week	Number of cases in the original data set (%)	Number of cases in the independent test set (%)
28	30 (11.9)	5 (12.5)
29	30 (11.9)	3 (7.5)
30	30 (11.9)	4 (10.0)
31	21 (8.3)	4 (10.0)
32	22 (8.7)	3 (7.5)
33	24 (9.5)	5 (12.5)
34	28 (11.1)	5 (12.5)
35	17 (6.7)	3 (7.5)
36	17 (6.7)	4 (10.0)
37	22 (8.7)	2 (5.0)
38	11 (4.4)	2 (5.0)
	252	40

**Table 2:** Characteristics of the study cohort.

Variables	Original data set (mean ± SD or n (%))	Independent test set (mean ± SD or n (%))
No. of cases	252	40
Maternal age(years)	29.7 ± 4.0	30.1 ± 3.7
Gestational age at delivery (wks)	39.1 ± 1.2	38.57 ± 1.1
Birth weight (g)	3336.4 ± 344.9	3304.9 ± 367.3
Birth height(cm)	49.8 ± 3.2	49.9 ± 1.7
vaginal delivery	114 (45.2%)	17 (42.5%)
Cesarean delivery	138 (54.8%)	23 (57.5%)

SD, standard deviation.

**Table 3** presents the regression analysis results for radiomic features derived from 2D and 3D ROIs. Features extracted from 3D ROIs yielded favorable estimates of gestational age in the independent test set: SVR (R²: 0.689, MAE: 1.290) and PLSR (R²: 0.710, MAE: 1.236). The standard deviation of all metrics across the training, validation, and independent test sets was consistently low, particularly in the independent test set, where SVR (R²: ± 0.014, MAE: ± 0.068) and PLSR (R²: ± 0.021, MAE: ± 0.056) demonstrated high model stability. Conversely, features extracted from 2D ROIs yielded significantly poorer estimates. Performance was already suboptimal in the training set: SVR (R² = 0.519) and PLSR (R² = 0.435). In the independent test set, the models exhibited poor predictive performance, characterized by negative R² values for both SVR (R²= −9.446) and PLSR (R² = −6.327). Regarding Pearson correlation coefficients (r), the 3D ROI models demonstrated strong performance, achieving r values of 0.904 (SVR) and 0.902 (PLSR) in the original dataset, and 0.846 (SVR) and 0.861 (PLSR) in the independent test set. The estimation results obtained using the PLSR model were comparable with those achieved with the SVR model.

**Table 3:** Estimated results of regression analyses.

Dataset	Metric	3D		2D	
		SVR	PLSR	SVR	PLSR
Training Set	R^2^	0.826 ± 0.005	0.830 ± 0.005	0.519 ± 0.061	0.435 ± 0.0137
		(0.815,0.832)	(0.818,0.836)	(0.431, 0.600)	(0.419,0.459)
	Adjusted−R^2^	0.821 ± 0.005	0.824 ± 0.006	0.505 ± 0.006	0.406 ± 0.015
		(0.809,0.827)	(0.813,0.831)	(0.401,0.579)	(0.389,0.431)
	MAE	0.992 ± 0.019	1.002 ± 0.019	1.596 ± 0.183	1.892 ± 0.030
	MSE	1.628 ± 0.045	1.591 ± 0.047	4.509 ± 0.586	5.293 ± 0.137
	RMSE	1.276 ± 0.018	1.261 ± 0.019	2.119 ± 0.137	2.301 ± 0.030
Validation Set	R^2^	0.816 ± 0.049	0.810 ± 0.049	0.346 ± 0.102	0.378 ± 0.142
		(0.770,0.937)	(0.749,0.930)	(0.170,0.485)	(0.179,0.552)
	Adjusted−R^2^	0.741 ± 0.073	0.736 ± 0.075	-0.211 ± 0.195	-0.149 ± 0.276
		(0.676,0.911)	(0.645,0.901)	(-0.532,0.050)	(-0.516,0.174)
	MAE	1.042 ± 0.168	1.047 ± 0.173	2.047 ± 0.182	1.983 ± 0.267
	MSE	1.721 ± 0.046	1.768 ± 0.452	6.103 ± 1.039	5.839 ± 1.394
	RMSE	1.295 ± 0.207	1.316 ± 0.193	2.461 ± 0.212	2.399 ± 0.289
Independent test set	R^2^	0.689 ± 0.014	0.710 ± 0.021	-9.446 ± 10.509	-6.327 ± 0.627
		(0.675,0.712)	(0.660,0.728)	(-42.053,-3.363)	(-7.322,-5.317)
	Adjusted−R^2^	0.619 ± 0.018	0.643 ± 0.028	-14.175 ± 16.268	-9.313 ± 0.930
		(0.601,0648)	(0.584,0.666)	(-59.594,-5.140)	(-10.713,-7.890)
	MAE	1.290 ± 0.068	1.236 ± 0.056	7.478 ± 3.071	7.225 ± 0.313
	MSE	2.722 ± 0.012	2.550 ± 0.190	91.385 ± 91.955	64.111 ± 5.486
	RMSE	1.649 ± 0.037	1.592 ± 0.057	8.893 ± 3.507	8.000 ± 0.342

Values are presented as Mean ± Standard Deviation (SD).

**Table 4** lists the best-performing gestational age–related features derived from 2D and 3D ROIs. A significant difference was observed between the two feature sets, with “MajorAxisLength” being the only common feature.

**Table 4:** The best set of features related to gestational age.

Modality	Feature type	Feature	Weight
3D	3D Shape	MajorAxisLength	2.7842
		MinorAxisLength	0.9652
	First Order	InterquartileRange	−0.3909
	GLCM	Correlation	0.2238
	GLRLM	GrayLevelNonUniformity	−0.5207
		ZoneVariance	−0.2848
	NGTDM	Busyness	−0.3785
2D	2D Shape	Elongation	−0.1425
		MajorAxisLength	1.4404
		MaximumDiameter	0.3607
		phericity	−0.5274
	First Order	Minimum	−0.3129
	GLCM	ClusterShade	−0.282
		MCC	−0.2753
	GLDM	DependenceEntropy	0.6479
		LowGrayLevelEmphasis	−0.4678
	GLSZM	GrayLevelNonUniformity	−1.2336
		SmallAreaEmphasis	0.101

Weights represent mean coefficients from 10-fold LASSO regression.

**Figure 3:**
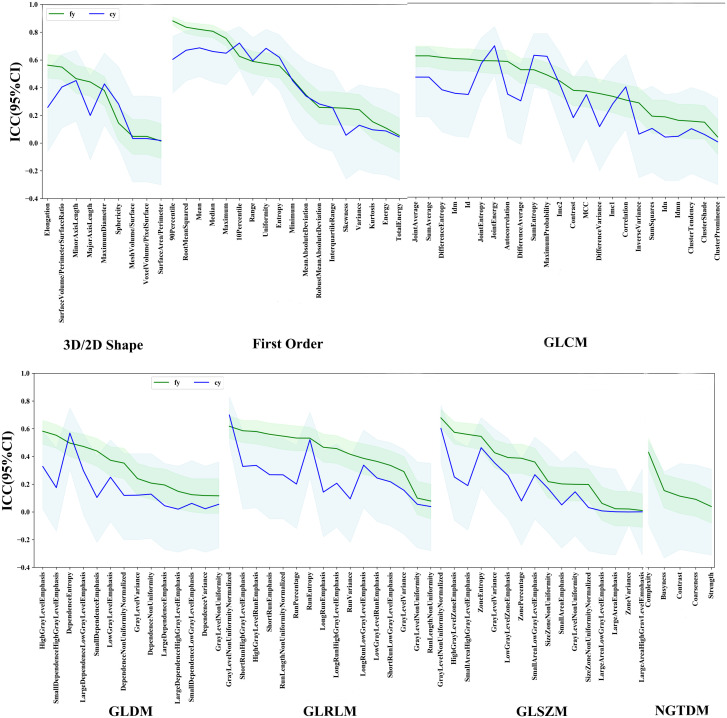
Intraclass correlation coefficients (ICCs) and 95% confidence intervals (95% CIs) for radiomics features extracted from 2D and 3D ROIs of the two datasets. Green lines and ribbons represent ICCs and 95% CIs for the original dataset, respectively. Blue lines and ribbons represent ICCs and 95% CIs for the independent test set, respectively. Features are grouped by class.

**Figure 3** illustrates the agreement between radiomic features derived from 2D and 3D ROIs. In the original dataset, the median ICC was 0.379 (interquartile range IQR: 0.179–0.554; range: 0.010–0.880). Specifically, five (4.9%) features demonstrated good agreement, 29 (28.4%) showed moderate agreement, and 68 (66.7%) displayed poor agreement. Notably, all five features with good agreement belonged to the ‘first-order’ class. In the independent test set, the median ICC of all features was 0.247 (IQR: 0.060–0.384; range: −0.024–0.721). Within this set, no features demonstrated good agreement, 17 (16.7%) exhibited moderate agreement, and 85 (83.3%) showed poor agreement.

The inter-rater reliability among the three radiologists for the 3D segmentations was excellent, with a median ICC of 0.956 across all radiomics features. Complete results are available in **Supplementary Material S1**.

## Discussion

Previous studies have explored various methods to assess lung development, including measuring fetal growth parameters, evaluating pulmonary circulation with Doppler ultrasound, and calculating the lung-to-liver signal intensity ratio on MRI (Moshiri et al., 2013). In this context, lung volume measurement has been suggested as a superior method for assessing lung development (Büsing et al., 2008; Ogawa et al., 2018; Prayer et al., 2023). In clinical practice, lung volume—or the ratio of measured to predicted lung volume—is often used to predict outcomes in fetuses with conditions such as congenital diaphragmatic hernia or congenital pulmonary airway malformations. However, the significant variability in lung volume values, particularly in late pregnancy (Ogawa et al., 2018), underscores the need for more accurate and robust methods.

Cobo et al. (2012) demonstrated that quantitative texture features extracted from ultrasound images of fetal lungs using AQUA software could effectively estimate gestational age. This finding has spurred extensive research, with several teams utilizing texture analysis for predicting neonatal respiratory morbidity (Bonet-Carne et al., 2015; Palacio et al., 2017; Burgos-Artizzu et al., 2019; Du et al., 2022). However, a notable limitation inherent in these studies—and indeed in many 2D imaging analyses—is the reliance on a single-plane image (typically at the four-chamber view) to represent the entire organ. This restricted sampling fails to capture the comprehensive volumetric heterogeneity of the developing lung, providing a compelling rationale for the adoption of 3D-based radiomic approaches.

In contrast, MRI enables the acquisition of 3D image data of the entire fetal lung, facilitating more comprehensive analyses. Watzenboeck et al. (2023) observed that features extracted from 2D ROIs in fetal lung MRI were less representative of the entire organ than those derived from 3D ROIs. Building on this insight—and to our knowledge, as the first study to leverage both 2D and 3D radiomic features from fetal lung MRI for gestational age estimation—our results further validate the superiority of 3D models: across the training, validation, and independent test sets, 3D-based models consistently outperformed their 2D counterparts in terms of gestational age prediction accuracy. This superior performance is directly supported by our quantitative analysis: the agreement between radiomic features derived from 2D and 3D ROIs—reflecting the concordance when the same features are extracted from a single 2D plane versus the entire 3D volume—was poor (median ICCs: 0.379 and 0.247 in the original and independent test sets, respectively). This objectively demonstrates that a single 2D plane cannot adequately represent the complex, heterogeneous 3D structure of the developing lung.

Furthermore, our semi-automated 3D segmentation protocol itself was highly reproducible, with excellent inter-rater reliability (median ICC = 0.956). This confirms that the model’s superior performance relies on both comprehensive 3D data and a robust segmentation foundation.

Notably, consistent with Watzenboeck et al. (2023), we observed that first-order features exhibited the highest level of agreement between 2D and 3D ROIs. However, these specific features were not among the most influential for gestational age estimation in our models. This suggests that even the most ‘consistent’ 2D features may fail to capture the biologically relevant 3D information necessary for accurate developmental assessment, providing an additional, task-specific explanation for the suboptimal performance of the 2D models.

Among the extracted radiomic features, ‘MajorAxisLength’ demonstrated the highest importance score for gestational age estimation and was identified in both 2D and 3D ROI analyses. As a shape-based feature, this finding underscores the significant influence of fetal lung geometry on gestational age assessment. This result aligns with established clinical practice, where fetal growth trajectories—particularly linear dimensions—serve as critical indicators for monitoring lung growth and development throughout pregnancy (Johnsen et al., 2006; Leung et al., 2008).

The external validation provided a rigorous test of the model’s clinical generalizability. As anticipated in real-world scenarios, the estimation performance on the independent test set was lower than on the training set, with this degradation being markedly more pronounced in the 2D model than in the 3D model. This performance gap is largely attributable to the technical heterogeneity between the two institutions. Specifically, differences in MRI hardware and proprietary reconstruction algorithms can result in inherent discrepancies in image resolution and background noise. Furthermore, variations in acquisition parameters—such as slice thickness and field of view (FOV)—directly dictate the level of structural detail captured, while divergent technical preprocessing methods (e.g., interpolation and resampling) significantly alter voxel intensity distributions and image smoothness. Collectively, these factors introduce non-biological technical noise that compromises the stability of radiomic features (Zwanenburg et al., 2020; Hajianfar et al., 2024; Hodneland et al., 2024; Wennmann et al., 2024). Our observation that 3D radiomic features exhibited greater robustness against this inter-site variability supports the premise that features derived from the entire organ volume are inherently less susceptible to these site-specific technical nuances compared to those derived from a single 2D plane.

This study has several limitations that warrant consideration. First, although we included an independent external validation set, the sample size (n=40) remained relatively small, and the distribution of cases across gestational weeks was not entirely balanced. This necessitates caution when generalizing our findings to the broader population across all gestational stages. Second, as discussed, technical heterogeneity between institutions remains a confounding factor. Variations in MRI hardware, acquisition parameters, and preprocessing methods can impact radiomic feature stability. While we employed Z-score normalization to mitigate these effects, the residual inter-site variability underscores the necessity for future multi-institutional studies that prioritize standardized imaging protocols and robust harmonization techniques. Third, to achieve an adequate sample size, we included cases where the primary focus of the scan was not lung-centric (e.g., head or spine scans), which may have introduced variability in image quality compared to protocols optimized for lung imaging. Finally, while our models effectively utilized MajorAxisLength, we did not formally disentangle its predictive contribution from texture features. Future work should address this through head-to-head comparisons with simple geometric metrics to clearly quantify the added value of complex radiomic signatures.

## Conclusions

In conclusion, this study demonstrates that 3D radiomic features offer superior performance in gestational age estimation compared to 2D approaches, establishing them as a more robust biomarker for fetal lung development. Translating this approach into clinical practice requires standardized image acquisition and processing pipelines to ensure model generalizability across institutions. These findings lay a critical foundation for applying 3D radiomics to high-risk populations—such as fetuses with congenital diaphragmatic hernia or pulmonary hypoplasia—potentially enabling more personalized and precise prenatal management.

## Data Availability

The raw data supporting the conclusions of this article will be made available by the authors, without undue reservation.
