# Supplementary material for: Quantitative assessment of fetal lung development using MRI-based radiomics: a comparative analysis of 3D and 2D approaches with external validation
Source: Front Physiol. 2026 Jul 29;17:1920572. doi: 10.3389/fphys.2026.1920572 (PMC13461516; doi:10.3389/fphys.2026.1920572)
Supplement: Supplementary file 1 [file DataSheet1.doc]

****Supplementary Material S1: Inter-rater Reliability of 3D Lung Segmentation****

****Purpose****

To evaluate the inter-observer reproducibility of the manual 3D lung segmentation protocol, which is the critical first step for robust radiomics feature extraction in this study.

****Methods****

A random subset of 25 cases was selected from the original cohort. Three junior radiologists, blinded to each other’s work, independently performed 3D lung segmentation following the standardized protocol. For each of the 102 radiomics features subsequently extracted, inter-rater reliability was quantified using the intraclass correlation coefficient (ICC) based on a two-way random-effects model for absolute agreement of single measurements (ICC(2,1)).

****Results****

Inter-rater reliability was excellent. The overall median ICC was 0.956 (interquartile range [IQR]: 0.870–0.982). As summarized below, 95.1% of features (97/102) demonstrated good-to-excellent reliability (ICC ≥ 0.75), and no feature exhibited poor reliability (ICC < 0.50). Reliability was consistently high across all feature categories.

| **Table S1-A. Summary of Inter-rater Reliability** | |
| --- | --- |
| Metric | Result |
| Overall Median ICC (IQR) | 0.956 (0.870–0.982) |
| Features with Excellent Reliability (ICC ≥ 0.90) | 68.6% (70/102) |
| Features with Good Reliability (0.75 ≤ ICC < 0.90) | 26.5% (27/102) |
| Features with Moderate Reliability (ICC < 0.75) | 4.9% (5/102) |
| Features with Poor Reliability (ICC < 0.50) | 0% (0/102) |

*Complete category-wise statistics are provided in Supplementary Table S1-B. The distribution of all ICC values is visualized in Supplementary Figure S1.*

****Conclusion****
This analysis confirms ****excellent reproducibility**** of the manual 3D segmentation protocol, thereby validating the foundation of the radiomics feature set used for predictive modeling in the main study.

Figure S1: Distribution of ICC(2,1) values across radiomics feature categories. The dashed lines indicate thresholds for good (0.75) and moderate (0.50) reliability. Each point represents an individual feature.

**Supplementary Table S1-B. ICC values for all 102 radiomics features**

| Feature | Category | ICC | 95% CI | Agreement |
| --- | --- | --- | --- | --- |
| TotalEnergy | First-order | 0.988 | 0.975–0.994 | Excellent |
| Uniformity | First-order | 0.984 | 0.966–0.993 | Excellent |
| 90Percentile | First-order | 0.979 | 0.959–0.991 | Excellent |
| Entropy | First-order | 0.976 | 0.951–0.989 | Excellent |
| InterquartileRange | First-order | 0.975 | 0.946–0.989 | Excellent |
| RobustMeanAbsoluteDeviation | First-order | 0.974 | 0.947–0.989 | Excellent |
| Median | First-order | 0.973 | 0.947–0.988 | Excellent |
| RootMeanSquared | First-order | 0.972 | 0.944–0.987 | Excellent |
| Mean | First-order | 0.971 | 0.943–0.987 | Excellent |
| MeanAbsoluteDeviation | First-order | 0.966 | 0.932–0.985 | Excellent |
| Energy | First-order | 0.962 | 0.924–0.983 | Excellent |
| 10Percentile | First-order | 0.958 | 0.918–0.981 | Excellent |
| Variance | First-order | 0.951 | 0.903–0.978 | Excellent |
| Maximum | First-order | 0.858 | 0.739–0.933 | Good |
| Skewness | First-order | 0.847 | 0.721–0.927 | Good |
| Range | First-order | 0.824 | 0.683–0.916 | Good |
| Minimum | First-order | 0.774 | 0.605–0.890 | Good |
| Kurtosis | First-order | 0.743 | 0.558–0.873 | Moderate |
| MaximumProbability | GLCM | 0.995 | 0.990–0.998 | Excellent |
| JointEnergy | GLCM | 0.991 | 0.982–0.996 | Excellent |
| Idm | GLCM | 0.991 | 0.981–0.996 | Excellent |
| InverseVariance | GLCM | 0.991 | 0.981–0.996 | Excellent |
| Id | GLCM | 0.991 | 0.981–0.996 | Excellent |
| JointEntropy | GLCM | 0.985 | 0.970–0.994 | Excellent |
| DifferenceEntropy | GLCM | 0.985 | 0.969–0.993 | Excellent |
| DifferenceAverage | GLCM | 0.985 | 0.969–0.993 | Excellent |
| SumEntropy | GLCM | 0.979 | 0.957–0.990 | Excellent |
| Contrast | GLCM | 0.976 | 0.952–0.989 | Excellent |
| DifferenceVariance | GLCM | 0.966 | 0.934–0.985 | Excellent |
| SumSquares | GLCM | 0.961 | 0.923–0.982 | Excellent |
| ClusterTendency | GLCM | 0.953 | 0.909–0.979 | Excellent |
| Imc2 | GLCM | 0.945 | 0.894–0.975 | Excellent |
| Imc1 | GLCM | 0.935 | 0.874–0.970 | Excellent |
| Correlation | GLCM | 0.932 | 0.869–0.969 | Excellent |
| ClusterProminence | GLCM | 0.882 | 0.779–0.945 | Good |

*Continued on next page*

| Feature | Category | ICC | 95% CI | Agreement |
| --- | --- | --- | --- | --- |
| Autocorrelation | GLCM | 0.867 | 0.753–0.937 | Good |
| JointAverage | GLCM | 0.855 | 0.733–0.931 | Good |
| SumAverage | GLCM | 0.855 | 0.733–0.931 | Good |
| MCC | GLCM | 0.800 | 0.643–0.903 | Good |
| ClusterShade | GLCM | 0.778 | 0.609–0.892 | Good |
| Idmn | GLCM | 0.755 | 0.576–0.879 | Good |
| Idn | GLCM | 0.745 | 0.561–0.873 | Moderate |
| DependenceVariance | GLDM | 0.991 | 0.983–0.996 | Excellent |
| LargeDependenceEmphasis | GLDM | 0.991 | 0.981–0.996 | Excellent |
| DependenceNonUniformityNormalized | GLDM | 0.981 | 0.961–0.991 | Excellent |
| SmallDependenceEmphasis | GLDM | 0.978 | 0.955–0.990 | Excellent |
| DependenceNonUniformity | GLDM | 0.972 | 0.944–0.987 | Excellent |
| DependenceEntropy | GLDM | 0.964 | 0.928–0.984 | Excellent |
| GrayLevelNonUniformity | GLDM | 0.952 | 0.907–0.978 | Excellent |
| GrayLevelVariance | GLDM | 0.951 | 0.904–0.978 | Excellent |
| LargeDependenceLowGrayLevelEmphasis | GLDM | 0.920 | 0.847–0.963 | Excellent |
| SmallDependenceHighGrayLevelEmphasis | GLDM | 0.902 | 0.814–0.954 | Excellent |
| HighGrayLevelEmphasis | GLDM | 0.871 | 0.761–0.939 | Good |
| LargeDependenceHighGrayLevelEmphasis | GLDM | 0.822 | 0.680–0.915 | Good |
| LowGrayLevelEmphasis | GLDM | 0.791 | 0.630–0.898 | Good |
| SmallDependenceLowGrayLevelEmphasis | GLDM | 0.618 | 0.389–0.800 | Moderate |
| RunLengthNonUniformityNormalized | GLRLM | 0.989 | 0.978–0.995 | Excellent |
| ShortRunEmphasis | GLRLM | 0.989 | 0.976–0.995 | Excellent |
| RunPercentage | GLRLM | 0.988 | 0.976–0.995 | Excellent |
| LongRunEmphasis | GLRLM | 0.986 | 0.971–0.994 | Excellent |
| RunVariance | GLRLM | 0.985 | 0.969–0.993 | Excellent |
| GrayLevelNonUniformityNormalized | GLRLM | 0.983 | 0.964–0.992 | Excellent |
| RunEntropy | GLRLM | 0.970 | 0.940–0.987 | Excellent |
| RunLengthNonUniformity | GLRLM | 0.967 | 0.936–0.985 | Excellent |
| GrayLevelNonUniformity | GLRLM | 0.953 | 0.909–0.979 | Excellent |
| GrayLevelVariance | GLRLM | 0.950 | 0.903–0.977 | Excellent |
| ShortRunHighGrayLevelEmphasis | GLRLM | 0.875 | 0.767–0.941 | Good |
| HighGrayLevelRunEmphasis | GLRLM | 0.872 | 0.762–0.940 | Good |
| LongRunHighGrayLevelEmphasis | GLRLM | 0.862 | 0.746–0.935 | Good |
| LongRunLowGrayLevelEmphasis | GLRLM | 0.826 | 0.686–0.916 | Good |
| LowGrayLevelRunEmphasis | GLRLM | 0.789 | 0.628–0.897 | Good |
| ShortRunLowGrayLevelEmphasis | GLRLM | 0.779 | 0.613–0.892 | Good |
| ZoneVariance | GLSZM | 0.998 | 0.996–0.999 | Excellent |

*Continued on next page*

| Feature | Category | ICC | 95% CI | Agreement |
| --- | --- | --- | --- | --- |
| LargeAreaEmphasis | GLSZM | 0.998 | 0.996–0.999 | Excellent |
| LargeAreaLowGrayLevelEmphasis | GLSZM | 0.984 | 0.967–0.993 | Excellent |
| ZonePercentage | GLSZM | 0.983 | 0.964–0.992 | Excellent |
| SizeZoneNonUniformity | GLSZM | 0.972 | 0.945–0.987 | Excellent |
| GrayLevelNonUniformityNormalized | GLSZM | 0.970 | 0.941–0.987 | Excellent |
| GrayLevelNonUniformity | GLSZM | 0.969 | 0.939–0.986 | Excellent |
| ZoneEntropy | GLSZM | 0.948 | 0.897–0.977 | Excellent |
| GrayLevelVariance | GLSZM | 0.946 | 0.895–0.975 | Excellent |
| SmallAreaEmphasis | GLSZM | 0.925 | 0.854–0.966 | Excellent |
| SizeZoneNonUniformityNormalized | GLSZM | 0.923 | 0.851–0.965 | Excellent |
| LargeAreaHighGrayLevelEmphasis | GLSZM | 0.915 | 0.837–0.960 | Excellent |
| SmallAreaHighGrayLevelEmphasis | GLSZM | 0.888 | 0.789–0.947 | Good |
| HighGrayLevelZoneEmphasis | GLSZM | 0.881 | 0.779–0.944 | Good |
| LowGrayLevelZoneEmphasis | GLSZM | 0.795 | 0.638–0.901 | Good |
| SmallAreaLowGrayLevelEmphasis | GLSZM | 0.764 | 0.590–0.884 | Good |
| Coarseness | NGTDM | 0.982 | 0.963–0.992 | Excellent |
| Contrast | NGTDM | 0.954 | 0.910–0.979 | Excellent |
| Complexity | NGTDM | 0.940 | 0.883–0.972 | Excellent |
| Strength | NGTDM | 0.770 | 0.597–0.887 | Good |
| Busyness | NGTDM | 0.738 | 0.549–0.870 | Moderate |
| MajorAxisLength | Shape | 0.998 | 0.995–0.999 | Excellent |
| MeshVolume | Shape | 0.992 | 0.983–0.996 | Excellent |
| MinorAxisLength | Shape | 0.986 | 0.972–0.994 | Excellent |
| SurfaceArea | Shape | 0.984 | 0.952–0.994 | Excellent |
| SurfaceVolumeRatio | Shape | 0.962 | 0.894–0.985 | Excellent |
| Elongation | Shape | 0.928 | 0.861–0.967 | Excellent |
| Sphericity | Shape | 0.870 | 0.650–0.949 | Good |
| Flatness | Shape | 0.846 | 0.654–0.934 | Good |
| Maximum3DDiameter | Shape | 0.551 | 0.306–0.758 | Moderate |
