# Supplementary material for: Quantitative assessment of fetal lung development using MRI-based radiomics: a comparative analysis of 3D and 2D approaches with external validation
Source: Front Physiol. 2026 Jul 29;17:1920572. doi: 10.3389/fphys.2026.1920572 (PMC13461516; doi:10.3389/fphys.2026.1920572)
Supplement: Supplementary file 2 [file DataSheet2.doc]

****Table S2 The main SVR and PLSR parameters for GridSearchCV.****

| method | parameter |  |
| --- | --- | --- |
| SVR | kernel | 'linear', 'rbf', 'poly', 'sigmoid' |
|  | C | [0.01,0.1,1,5,10] |
|  | degree | [3,8] |
|  | coef0 | [0.01,0.5,1,5,10] |
|  | gamma | 'auto','scale' |
| PLSR | n_components | [1,2,3,4,5,6,7,8] |
